# Effects of a one-week vacation with various activity programs on well-being, heart rate variability, and sleep quality in healthy vacationers—an open comparative study

**DOI:** 10.1186/s12889-022-14838-7

**Published:** 2022-12-27

**Authors:** Markus Hübner, Peter Lechleitner, Günther Neumayr

**Affiliations:** 1 Tirol Kliniken, Innsbruck, Austria; 2 Symbiomed Medical Center Lienz, and Department of Internal Medicine, Hospital Lienz, Lienz, Austria; 3 Medical Office of Dr. Neumayr, Lienz, Austria

**Keywords:** Physical activity, Vacation, Psychological well-being, Stress, Heart rate variability, Sleep quality

## Abstract

**Objectives:**

This open comparative study aimed to analyze the effects of a one-week vacation with various activity programs on well-being, heart rate variability (HRV) and sleep quality in healthy vacationers.

**Methods:**

Fifty-two healthy untrained vacationers spent a one-week vacation with regular exercise in East Tyrol. Exercise was performed on six of seven days. The study participants were divided into a) Group 1, playing golf (G), and b) Group 2 performing Nordic walking or e-biking (NW&EB). Well-being was measured with the WHO-5 well-being-index; stress and recovery status was obtained with the EBF-24-questionnaire (recovery-stress questionnaire). HRV parameters in the time and frequency domain (SDNN, pNN50, r-MSSD, log LF/HF and total power) were measured with a 24-h-ECG (electrocardiogram). Sleep quality was derived from the EBF-24 questionnaire and sleep architecture from HRV-analysis. Examinations were performed one day before and after the vacation.

**Results:**

Well-being significantly improved in the G group (+ 40%, *p* < 0.001) and NW&EB group (+ 19%, *p* = 0.019). The stress and recovery profile also improved significantly in both groups (stress-decrease: -43.7% G group; -44.7% NW&EB group; recovery-increase: + 23.6% G group; + 21.5% NW&EB group). Except for the SDNN (standard deviation of the NN interval), no significant change was noted in HRV-parameters. SDNN improved significantly only in the NW&EB group (+ 9%, *p* < 0.05). Sleep quality (+ 21% G group, *p* = 0.029; + 19% NW&EB group, *p* = 0.007) and architecture (-10% G group, *p *= 0.034; -23% NW&EB group, *p* = 0.012) significantly improved in both groups.

**Conclusion:**

A short-term vacation with regular exercise was well tolerated by the study participants and improved well-being, sleep quality, HRV and autonomic regulation.

**Trial registration:**

Registry and the registration no. of the study/trial: Approval was received from the ethics committee of the Leopold Franzens University of Innsbruck (AN2013-0059 332/4.8).

## Introduction

Regular physical training has positive effects on both physical and mental health, supports the individual to achieve an ongoing reduction of daily stress, and is recommended worldwide as a beneficial lifestyle measure by various health organization [1–3]. Vacation, defined by Lounsbury and Hoopes in 1986 as a temporary period of several days or weeks off from work, is also considered “healthy” for recovery from work stress, although scientific data in this regard are scarce. Scientific research investigating the impact of vacation and exercise therein on medical risk factors or established health parameters is virtually non-existent [4–6]. The cardiovascular and metabolic part of the OGTS study has shown that physical exercise during a vacation significantly reduces medical risk factors such as diastolic blood pressure, blood sugar and adipokine levels [4, 6]. In Western societies holiday behavior reveals a tendency towards short vacations. The question arises as to whether short-term vacations provide sufficient recovery from work and everyday stress [7].

A chronic unstable balance between stress and recovery will cause exhaustion sooner or later; Vacation constitutes a longer break from work and may be regarded as a form of “macro regeneration “ preventing a chronic unstable balance between stress and recovery, which cannot be compensated by a weekend or a few days off from work [8]. Any chronic overload situation, regardless of whether it is a burnout syndrome at the emotional-psychological level or an overtraining syndrome at the physical level, causes an alteration of HRV parameters [9]. Regular physical training above an individual threshold causes physiological adaptations in all organ systems, increases parasympathetic function overnight including autonomic regulation of the cardiac system [10].

To derive a healthy or pathological sleep architecture from HRV analysis, attention is paid to the nocturnal respiratory sinus arrhythmia (RSA) in addition to the tub-shaped lowering of the heart rate indicating preserved parasympathetic activity. The occurrence of apnea episodes and so-called "arousals" are also registered [3, 11–13]. For a pathologic sleep architecture increased nocturnal sympathetic activity appears to be the trigger, counteracting physiological parasympathetic predominance during sleep [14]. Previous studies found a significant improvement in sleep quality, sleep duration and well-being during vacation. The results were even more pronounced in study participants performing physical activities during their vacation [15–17].

The study design of the East Tyrol Health Tourism Study (OGTS) was a prospective, open comparative study on the effects of a one-week structured holiday stay in East Tyrol with different activity programs (Nordic walking, golfing, e-biking) on circulatory, metabolic and regeneration parameters. According to our inclusion criteria we selected healthy adult participants (age: 35—65 years, equal male:female ratio in the groups, Vo2max power range: 25–45 ml/min/kg, BMI 20–35), who had no shortness of breath, angina pectoris or other complaints during moderate exercise (> 5 MET`s), featuring a stable blood pressure and a heart rate below 100/min in sinus rhythm.

All sports activities – golf, Nordic walking and e-biking – were of low to moderate exercise intensities and could be performed by nearly everyone, including untrained persons. The parameters investigated in this part of the OGTS, i.e. psychological well-being, HRV, and sleep quality are influenced negatively by any kind of stress and positively by the quantity of regeneration and physical activity. The purpose of the OGTS was to investigate the cumulative health effects of a one-week vacation and regular physical activity with regard to three hypotheses:

- Hypothesis 1 (H1): A short-term vacation with moderately intensive sports activities has measurable and positive effects on psychological well-being.
- Hypothesis 2 (H2): HRV is positively influenced by a week of active vacation.
- Hypothesis 3 (H3): The quality of sleep is improved during a vacation.

## Materials and methods

### Design and procedure

The present study was part of the East Tyrol Health Tourism Study (OGTS) and investigated the effects of a short-term vacation with activity programs on psychological well-being, and various autonomic functions such as HRV and sleep quality in healthy volunteers. The cardiovascular and metabolic findings of the OGTS—describing the effects on performance capacity, blood pressure, heart rate and diastolic heart function as well as changes in metabolism and the adipokine profile—were published previously [4, 6].

In an open comparative study, 52 healthy untrained vacationers spent a one-week active vacation in East Tyrol. All participants provided their written consent prior to the study [4]. The study was approved by the ethics committee of the Medical University of Innsbruck. The study participants were accommodated at one of the eight hotels in East Tyrol, at an altitude of 670 to 980 m above sea level [4]. Three sports programs were available: golf, Nordic walking, and e-biking. Nordic walking and e-biking were combined in a single group because the exercise intensity and duration were similar. Group 1 played golf and group 2 performed Nordic walking or e-biking (NW&EB). Group assignment was based on the vacationer ‘s preference. The mean duration of exercise during an e-biking or walking tour was 2.4 h in the NW&EB group. The mean duration of exercise was 33.5 h/week in the G group and 14.2 h/week in the NW&EB group. Every training unit was guided by a fitness instructor. The participants’ daily caloric intake was not taken into account; a specific diet plan was not followed. Psychological well-being was registered on the WHO-5 well-being index, and the current stress and recovery profile with the EBF-24 questionnaire on stress and recovery. HRV analysis was performed on the basis of a 24-h-ECG evaluated in the Symbiomed Medical Center Lienz. Sleep quality was registered on the EBF-24 questionnaire, and sleep architecture was derived from the HRV analysis. All examinations were performed one day before and after the vacation. A second survey of the OGTS was carried out two years after the one-week vacation to examine long-term effects in terms of lifestyle modification, diet, physical activity, and the ability to relax. The protocol timeline is provided in (Table 1).

**Table 1:**
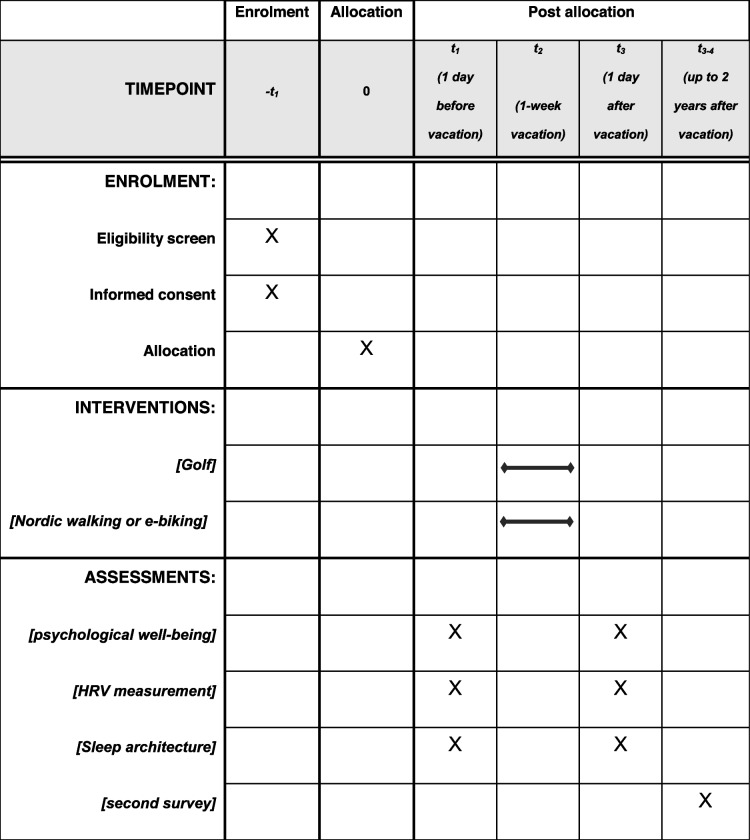
Spirit (standard protocol items: recommendations for interventional trials)—protocol timeline

### Measurements

#### Well-being

Psychological well-being was assessed with the German version of the original WHO-5 well-being index of 1998. The WHO-5 questionnaire includes positively phrased feelings such as a good mood, feeling calm and relaxed, active and vigorous, fresh and rested, and interested in matters of everyday life [18].

Results are expressed on a 6-point Likert scale for each item, ranging from 0 to 5: 0 = at no time, 1 = some of the time, 2 = less than half the time, 3 = more than half the time, 4 = most of the time, 5 = all of the time. This provides a prime score ranging from 0 to 25. The result is multiplied by 4 to obtain a standardized percentage of points, yielding a figure between 0 and 100%. A high score indicates a good result whereas a low score indicates a bad result [18].

#### Stress and recovery

The recovery-stress questionnaire (EBF) used in the present study was the German version of the EBF-Basic-24-A3 / B3. The EBF-24 questionnaire comprises 12 topics: 7 of these address stress and 5 address recovery. Each topic consists of 2 items [19, 20]. We evaluated 4 of 7 topics addressing stress: feelings such as dejection, irritability, tiredness and subjective malaise, and 4 of 5 subjects addressing recovery: feelings such as relaxation, mood, satisfaction, and sleep quality. Each of the 12 subjects consisting of 2 items was mapped on a 7-point Likert scale ranging from 0 (never) to 6 (always). A minimum of 0 points and a maximum 12 points can be reached in the summation for each subject. The questionnaire was modified to the current study design, and assessed the previous 7–14 days and nights A high score reveals a high stress level, whereas a low score reveals a low stress level. For recovery a high score reveals a good regeneration profile, whereas a low score implies insufficient recovery [19, 20].

#### HRV

HRV analysis was performed with a 24-h-ECG (Medilog AR12 plus®, Schiller, Feldkirchen, Germany) in the Medical Center of Grand Hotel Lienz. The 24-h ECG was evaluated with the Darwin software from Schiller and no additional manual correction was performed. HRV parameters were automatically evaluated via the Medilog AR 12 plus from Schiller providing precise HRV analysis with a sampling rate of approximately 4000/sec. The whole 24-h register was considered in the analysis.

The following parameters were measured: SDNN, r-MSSD, pNN50, total power and log LF/HF. SDNN: standard deviation of the NN intervals in ms; pNN50: percentage of the intervals with at least 50 ms deviation from the previous interval; r-MSSD: root mean square of successive differences between normal heartbeats; log LF/HF: quotient of the logarithm of low frequency to high frequency; total power: total number of all frequency ranges [21, 22].

#### Sleep quality

Sleep quality was investigated by the EBF-24 questionnaire, the apnea index, and HRV analysis. The following parameters were used to assess sleep architecture from HRV analysis: nocturnal, tub shaped heart rate depression, respiratory sinus arrhythmia (RSA) (ranging from 0.2 to 0.3 Hz), arousals, and periods of deep sleep [3, 11–13]. The deep sleep stage is characterized by increasing oscillations in the HF range—especially in the range of the RSA. A simultaneous reduction of the LF, VLF and ULF spectra and alteration of HF shows the predominance of the parasympathetic nervous system during this sleep stage [13]. The so-called tub-shaped drop in heart rate at night is part of the normal sleep architecture and is a feature of the predominant parasympathetic activity during rest and at night [11].

Apnea index: if the individual experiences episodes of apnea the organism is startled and experiences a so-called "arousal" of the sympathetic nervous system, with an increasing heart rate to compensate for the impending lack of oxygen [3, 11–13]. During an apnea, the oscillations in the LF spectrum increase especially at 0.05 Hz, indicating increased sympathetic activity [12].

We determined a score ranging from 1 to 4 for each study participant. The scoring was based on: appearance of episodes of apnea followed by an “arousal”, respiratory sinus arrhythmia (RSA), deep sleep stages and tub-shaped drop in heart rate. (1 = normal sleep architecture; 2 = slightly disturbed sleep architecture; 3 = moderately disturbed sleep architecture; 4 = strongly disturbed sleep architecture).

### Statistical analysis

IBM SPSS Statistics for Mactonish, Version 26.0 (IBM Corp., Armonk, N.Y., USA) was used for statistical analysis. Wilcoxon’s rank sum test was employed to determine *p-values* for the before-and-after comparison. The interquartile range (IQR, 25th and 75th percentiles) was also calculated. Cohens d_s_ was calculated to assess if the magnitude of variation was of low, medium, or high importance. A calculation of the sample size was performed with the G*Power software version 3.1 for each study group. The minimum sample size required for each study group with an effect size dz = 0.5, margin of error = 0.05 and power (1-ß) = 0.8, was 28 for the G group and 21 for the NW&EB group. The level of statistical significance was set to *p* < *0.05.*

## Results

Fifty-two persons participated in the study (30 men and 22 women). Their median age was 54.3 years. Baseline characteristics were similar in both groups. The sole significant difference was the mean exercise duration secondary to the study design. Table 2 summarizes the baseline characteristics of the study participants.

**Table 2:** Baseline characteristics of the study participants before the one-week vacation

Parameter	Golf group	NW&EB group
Number (n)	30	22
Age (years)	54 (47–63)	54 (47–60)
Gender (m, %)	53	64
BMI (kg/m^2^)	27 (23–30)	27 (22–30)
Mean exercise duration/week (h)	33.5	14.2

Results shown as medians and interquartile range (IQR)

### WHO-5 well-being index

Before the vacation, the mean score on the WHO-5 well-being index was 15.3 (± 4.49) in the G group and 16.45 (± 4.86) in the NW&EB group. After the vacation, an increase of 5.29 points was noted in the G group and 3.14 points in the NW&EB group. The WHO-5 well-being index increased to 20.59 (± 2.67) in the G group and 19.59 (± 3.13) in the NW&EB group. The standardized percentage values increased from 60 to 84% in the G group (p < 0.001, Cohens d_s_ = -1.38), and from 64 to 80% in the NW&EB group (*p* = 0.019, Cohens d_s_ = -0.77). This corresponds to a relative percentage increase of 40% in the G group and 19% in the NW&EB group. The increase in the WHO-5 well-being index observed in both groups implies a significant improvement of well-being after one week of active vacation.

### EBF-24

The questionnaire was divided into two parts: stress and recovery. A detailed group comparison of mean values is given in (Table 3).

**Table 3:** Heart rate variability, well-being and sleep quality in the golf and Nordic walking / e-biking groups before and after the vacation

Parameter	Golf group (*n* = 30)		NW&EB group (*n* = 22)	
	Before the vacation	After the vacation	Before the vacation	After the vacation
EBF-24 stress				
Dejection	2.89 (1–4)	1 (0–1.75) **	1.91 (0–3)	0.95 (0–1)*
Irritability	3.21 (2–4)	2 (1–2)***	3.23 (2–4.75)	1.55 (0–2)**
Tiredness	4.32 (2.5–5)	2.07 (0.25–3)**	3.27 (2–4.75)	2.05 (1–3)*
Subjective malaise	3.32 (1.5–4)	2.67 (1.25–4)	3.50 (2–5)	2.05 (1.25–2)**
EBF-24 recovery				
Happiness	6.37 (5.5–8)	7.87 (6.25–9)*	5.32 (3–8)	6.64 (5–8.75)**
Relaxation	6.89 (5.5–8)	9.10 (8–10)***	6.41 (4–8)	8.68 (8–10)***
Satisfaction	7.53 (6–9.5)	8.97 (8–10)***	7.77 (6.25–9.75)	8.59 (8–10)
Sleep quality	7.58 (7–9)	9.13 (8.25 – 11)*	7.55 (6 – 10)	8.95 (8 – 11)**
WHO-5	15.3 (12–18)	20.59 (18.25–22)****	16.45 (13.5–20)	19.59 (17.25 – 21.75)*
SDNN (ms)	41.74 (32.05–48.85)	40.88 (34.7–46.4)	45.67 (36–53)	49.67 (40 – 56)*
pNN50 (%)	1.87 (0.5–2.5)	3.23 (0.57–3.6)	2.74 (0.77–2.9)	3.67 (0.71 – 5.54)
r-MSSD (ms)	20.09 (12.6–19.6)	20.91 (12.8–24.3)	19.51 (16–21.4)	21.84 (17.7 – 26.7)
log LF/HF (%)	0.68 (0.59–0.82)	0.67 (0.54–0.79)	0.61 (0.5–0.7)	0.60 (0.44 – 0.73)
Total power (ms^2^)	2021 (1224–2619)	2375 (1341–2979)	3212 (2415–4111)	3555 (2468 – 4714)
Sleep architecture	2.44 (2–3)	2.20 (2–3)*	2.35 (2–3)	1.80 (1–2)*
Sleep quality (EBF-24)	7.58 (7–9)	8.68 (7–9)	7.55 (6–10)	8.95 (8–11)
Apnea index	7.04 (0.71 – 10.6)	5.32 (0.5–9.12)	7.64 (3–8)	7.84 (4.6–10.3)

Results shown as mean values rounded to two decimal places and interquartile range (IQR) in the golf vs. NW&EB groups. *NW&EB* Nordic walking and e-biking. *WHO-5* World Health Organization-5 index of well-being, *SDNN* standard deviation of the NN intervals in ms, *pNN50* percentage of the intervals with at least 50 ms deviation from the previous interval, *r-MSSD* root mean square of successive differences between normal heartbeats, *log LF/HF* quotient of the logarithm of low frequency to high frequency, *total power* total number of all frequency ranges, *Sleep architecture (HRV)*: evaluation of sleep quality based on heart rate variability, *Sleep quality (EBF-24)* evaluation of sleep quality based on the stress-recovery questionnaire, *Apnea index* number of cessations of breathing, *ms* milliseconds; *ms*^2^ milliseconds squared. *p-value* within the group: *vs*. before the vacation *p < *0.05*; *vs*. before the vacation **p < *0.01*; *vs*. before the vacation ***p < *0.005*; *vs*. before the vacation ****p < *0.001*

#### Stress

Perceived stress was reduced significantly after one week in both groups. The NW&EB group showed a significant point reduction of the mean values in all four subtests evaluated for stress – *dejection*, *irritability*, *tiredness* and *subjective malaise* – which corresponds to a reduction of stress. The group participants felt *less dejected* (before vacation: 1.91 ± 1.95 vs. after vacation: 0.95 ± 1.15; -50%, *p* = *0.044,* Cohens d_s_ = 0.59), *less irritable* (before vacation: 3.23 ± 2.23 vs. after vacation: 1.55 ± 1.78; -52%, *p* = *0.05,* Cohens d_s_ = 0.83), *less tired* (before vacation: 3.27 ± 2.65 vs. after vacation: 2.05 ± 1.46; -38%, *p* = *0.045,* Cohens d_s_ = 0.57) and *less uncomfortable* (before vacation: 3.50 ± 2.17 vs. after vacation: 2.05 ± 1.15; -42%, *p* = *0.006,* Cohens d_s_ = 0.84). Golfers, by comparison, achieved statistical significance in 3 of 4 subtests: *dejection* (before vacation: 2.89 ± 2.40 vs. after vacation: 1 ± 1.18; -65%, *p* = *0.003,* Cohens d_s_ = 1.08), *irritability* (before vacation: 3.21 ± 2.19 vs. after vacation: 2 ± 2.09; -38%, *p* = *0.004,* Cohens d_s_ = 0.57) and *tiredness* (before vacation: 4.32 ± 2.64 vs. after vacation: 2.07 ± 2.16; -52%, *p* = *0.08,* Cohens d_s_ = 0.96). No significant outcome was observed for *subjective malaise* (before vacation: 3.32 ± 2.45 vs. after vacation: 2.67 ± 1.87; -20%, *p* = *0.874,* Cohens d_s_ = 0.3). In a group comparison, the improvement in feelings such as *dejection*, *irritability* and *tiredness* were more pronounced in the G group than in the NW&EB group. The cumulative stress value, which comprised mean values for *dejection, irritability, tiredness* and *subjective malaise*, decreased by 6 points in the G group (from 13.73 to 7.73 points), corresponding to a percentage decrease of 43.7%. In the NW&EB group, this value decreased by 5.3 points (from 11.9 to 6.6 points) or 44.7%. This resulted in a decrease of ~ 44% for stress reduction in the entire group (G + NW&EB).

#### Recovery

In both groups, the recovery profile improved after one week. The increase in all four evaluated subtests was more significant in golfers. Group members felt *more satisfied*, *happier*, *more relaxed,* and *more rested* than they did before the holiday. The score achieved by golfers on the EBF-24 questionnaire for *satisfaction* increased from 7.53 ± 2.11 to 8.97 ± 1.85 points [relative percentage increase of 19% (*p* = *0.003*), Cohens d_s_ = -0.74]. The NW&EB group showed a measurable but non-significant increase of 11% in *satisfaction* (before vacation: 7.77 ± 2.35 vs. after vacation: 8.59 ± 1.89; *p* = *0.063,* Cohens d_s_ = -0.38). In both groups, the study participants felt *more relaxed* after their vacation. The average increase in *relaxation* was 32% in the G group and 35% in the NW&EB group (*p* < *0.005*). Improvements in (before vacation: 5.32 ± 2.70 vs. after vacation: 6.64 ± 2.19; + 25%, *p* = *0.007,* Cohens d_s_ = -0.53), *relaxation* (before vacation: 6.41 ± 2.29 vs. after vacation: 8.68 ± 1.92; + 35%, *p* = *0.001,* Cohens d_s_ = -1.08) and *sleep quality* (before vacation: 7.55 ± 2.60 vs. after vacation: 8.95 ± 1.92 + 19%, *p* = *0.007,* Cohens d_s_ = -0.62) were more pronounced in the NW&EB group than in the G group. The latter achieved significant increases in their scores for *happiness* (before vacation: 6.37 ± 1.93 vs. after vacation: 7.87 ± 2.02; + 24%, *p* = *0.021,* Cohens d_s_ = -0.76), *relaxation* (before vacation: 6.89 ± 2.27 vs. after vacation: 9.1 ± 1.72; + 32%, *p* = *0.002,* Cohens d_s_ = -1.13) and *sleep quality* (before vacation: 7.58 ± 2.54 vs. after vacation: 9.13 ± 2.28; + 21%, *p* = *0.029,* Cohens d_s_ = -0.65). The cumulative recovery value, which comprised the mean values for *satisfaction*, *happiness*, *relaxation* and *sleep quality*, resulted in a point increase of 6.7 (from 28.4 to 35.1 points) in the G group, which corresponds to a percentage increase of 23.6%. This value increased by 5.8 points (from 27 to 32.8 points) or 21.5% in the NW&EB group. The entire group (G + NW&EB) experienced an improvement of ~ 23% in regeneration and recovery.

#### HRV

There was a small but non-significant change in all measured HRV parameters, except for a significant improvement of SDNN in the NW&EB group. The changes were similar in both groups. Golf group: r-MSSD (before vacation: 20.09 ± 12.97 ms vs. after vacation: 20.91 ± 12.76 ms; Cohens d_s_ = -0.06), pNN50 (before vacation: 1.87 ± 2.03 ms vs. after vacation: 3.23 ± 5 ms; Cohens d_s_ = -0.35), log LF/HF (before vacation: 0.68 ± 0.22% vs. after vacation: 0.67 ± 0.26%; Cohens d_s_ = 0.03) and total power (before vacation: 2021 ± 1231 vs. after vacation: 2375 ± 1497 ms^2^; Cohens d_s_ = -0.18). NW&EB group: r-MSSD (before vacation: 19.51 ± 5.95 ms vs. after vacation: 12.97 ± 6.92 ms; Cohens d_s_ = -0.36), pNN50 (before vacation: 2.74 ± 3.26 ms vs. after vacation: 3.67 ± 3.99 ms; Cohens d_s_ = -0.25), log LF/HF (before vacation: 0.61 ± 0.16% vs. after vacation: 0.60 ± 0.20%; Cohens d_s_ = 0.05) and total power (before vacation: 3212 ± 1348 vs. after vacation: 3555 ± 1616 ms^2^; Cohens d_s_ = -0.23). HRV parameters improved slightly but non-significantly in terms of increased parasympathetic activity in both groups. The mean SDNN was high at the start of the vacation in the NW&EB group, and increased significantly from 45.67 ± 12.49 ms to 49.67 ± 12.25 ms, corresponding to a relative percentage increase of 9% (*p* < *0.05,* Cohens d_s_ = -0.32). Details of the HRV parameters are shown in (Table 3).

### Sleep quality based on the EBF-24 questionnaire, HRV analysis, and apnea index

#### Sleep architecture (HRV)

**Fig. 1:**
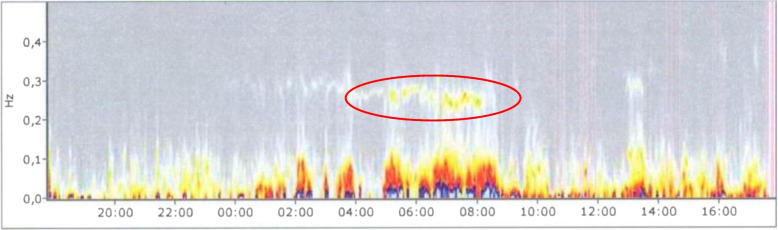
HRV spectrogram of a study participant one day before the vacation: The red ellipsoidal marking indicates a low frequency density in the HF spectrum (0.15–0.4 Hz), whereas the weak yellow coloring indicates reduced nocturnal PNS activity. X-axis: time of the HRV analysis; y-axis: frequency spectrum in Hz; Hz: Hertz; HF: high frequency. This spectrogram shows poor sleep quality and diminished nocturnal parasympathetic activity

**Fig. 2:**
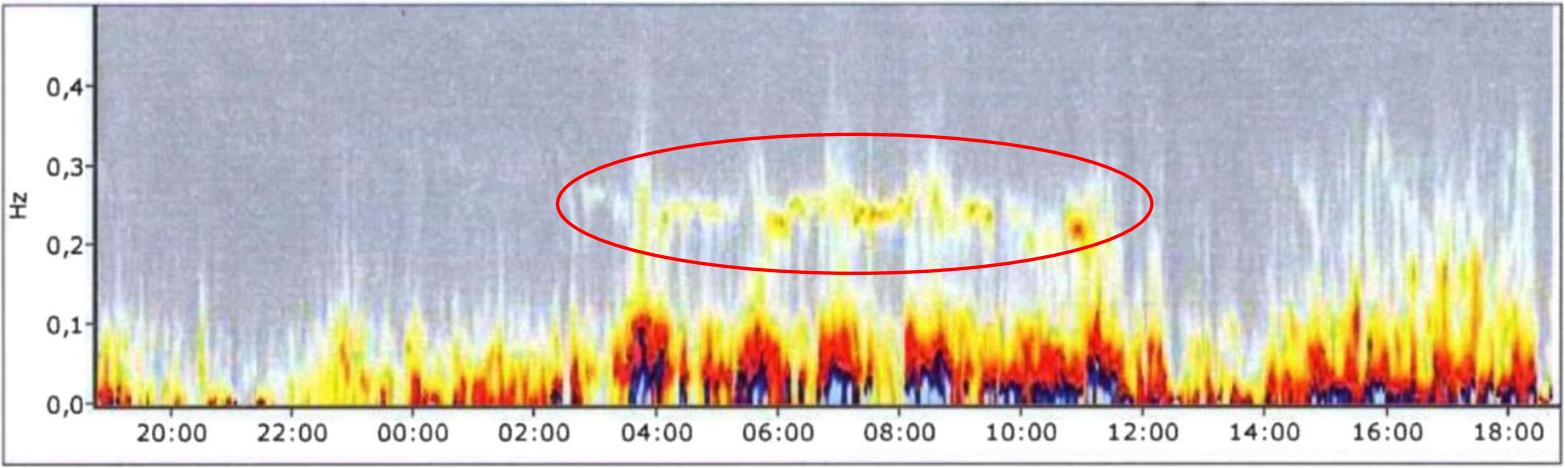
HRV spectrogram of the same study participant after the one-week vacation: The red ellipsoidal marking highlights the increased frequency density in the HF spectrum (0.15–0.4 Hz). Increasing yellow and light red coloring express good nocturnal parasympathetic activity. X-axis: time of the HRV analysis; y-axis: frequency spectrum in Hz; Hz: Hertz; HF: high frequency. This spectrogram shows improved sleep quality with demonstrable nocturnal parasympathetic activity

The score of the G group for sleep architecture was 2.44 ± 0.85 (on a scale from 1 to 4) before the vacation, and decreased to 2.2 ± 0.74 (-10%, *p* = *0.034,* Cohens d_s_ = 0.3) after the vacation. This result signifies an improvement from a *moderately* to a *slightly disturbed* sleep architecture. The decrease was even more pronounced in the NW&EB group. The mean value in this group decreased from 2.35 ± 0.79 to 1.8 ± 0.68 (-23%, *p* = *0.012*, Cohens d_s_ = 0.75). Sleep architecture changed significantly; it improved from a *moderately* to a *slightly disturbed* sleep architecture in the G group, and from a *slightly disturbed* to a *nearly normal* sleep architecture in the NW&EB group. A clear increase in nocturnal parasympathetic activity was noted on the HRV spectrogram. Figures 1 and 2 show changes in sleep architecture throughout the vacation in one study participant.

#### Sleep quality EBF-24

All study participants felt *more rested* in the morning and *less tired* after one week of active vacation compared to the beginning. The number of points on the EBF-24 questionnaire increased from 7.58 ± 2.54 to 9.13 ± 2.28 after the vacation in the G group (*p* = *0.029,* Cohens d_s_ = -0.65), and from 7.55 ± 2.60 to 8.95 ± 1.92 in the NW&EB (*p* = *0.007,* Cohens d_s_ = -0.62). The improvement of sleep quality was significant in both groups, but more pronounced in the NW&EB group.

#### Apnea index

The apnea index was calculated on the basis of the HRV analysis, and revealed no significant change in either group. The number of nocturnal apneas was only reduced among golfers (from.

6.66 ± 8.14 to 5.32 ± 5.94, Cohens d_s_ = 0.19). However, the apnea index increased in the NW&EB group (from 7.64 ± 7.98 to 7.84 ± 5.78, Cohens d_s_ = -0.029). Specific data on sleep quality are summarized in (Table 3).

## Discussion

The main results of the present study were the following:

1. A short vacation of 7 days with regular exercise was well tolerated by untrained study participants, may be considered safe, and significantly improved psychological well-being.
2. There was a positive effect on the global parasympathetic modulation, but it was moderate and mostly not significant.
3. Sleep quality and sleep architecture were also significantly improved.

*Ad H1)* Vacation is referred to as a form of “macro-regeneration” and is regarded as an effective form of regeneration to reduce accumulated stress better than a single evening or a weekend off from work [7]. Our study showed that a short-term vacation with various activity programs significantly improved well-being in both activity groups. The improvement was especially pronounced in the G group, whose members felt very stressed at the start of their vacation.

Our data were confirmed in a study published by Blank et al. in 2018, featuring a study design similar to the OGTS. Blank et al. described the effects of a short-term vacation on the stress level of German-speaking managers and observed positive effects of the vacation on well-being and strain reduction; the latter was more pronounced in the physically active intervention group [23]. The members of the intervention group spent four nights in a hotel and practiced various sports activities (Nordic walking or swimming and yoga or Qui-Gong) guided by a fitness instructor. The control group spent four nights at home, without working or exercising. Blank et al. noted significant reductions in strain levels by 1.3 score points in the intervention group and 0.9 in the control group. The increase in well-being and the strain reduction persisted for 45 days after the short-term vacation in both groups [23].

In our study, the stress level of the participants, evaluated with the EBF-24 questionnaire, was reduced by an average of 1.5 points in the G group and 1.3 points in the NW&EB group. We also registered positive long-term effects. The second survey of the OGTS, carried out two years later, showed that 85 percent of the study participants experienced positive long-term effects in terms of lifestyle modification, diet, physical activity, and the ability to relax.

Similar positive effects of a vacation on well-being were reported in a meta-analysis published by de Bloom et al. [7]. In this summary of several studies, which regrettably do not report the duration of the holiday in all cases and lack a structured activity program, the authors noted minor and rather temporary positive effects on health and well-being [7].

Regular exercise appears to be a decisive factor in improving well-being during a vacation. Our data show that regular exercise enhanced well-being to a significant extent in both study groups, regardless of the type of activity. This phenomenon was confirmed in a meta-analysis published by de Bloom et al. in 2012: the authors concluded that sporadic exercise (~ 27% of the vacation time) does not alter health parameters or well-being [5]. Our data suggest that the weekly duration of activity may exert a profound effect on the degree of improvement in well-being. The increase in well-being was more pronounced in the G group, whose mean exercise duration of 33.5 h per week was significantly longer than the exercise duration of 14.2 h per week in the NW&EB group. Our data appear to confirm the pre-existing notion that the regularity and duration of physical activity – and less so its intensity – improve well-being to a decisive extent [24, 25]. Our thesis is supported by an international meta-analysis published by Schuch et al. in 2018, which examined the relationship between physical activity and the lifetime risk of developing depression. This study showed that a higher level of physical activity is liable to reduce the lifetime risk of depression [24].

Thus, hypothesis 1, which stated that a short-term vacation with activities of moderate intensity has a positive impact on psychological well-being, was confirmed.

*Ad H2)* Our data showed that an active short-term vacation influenced HRV positively, although the changes were significant only in the NW&EB group. SDNN in the 24-h HRV analysis is the gold standard to stratify cardiovascular risk, and was proven to be a reliable prognostic parameter of morbidity and mortality in long-term studies. SDNN values below 50 ms are interpreted as a risk factor. Persons with values between 50 and 100 ms are considered somewhat restricted, and those with values above 100 ms are considered normal or healthy [21]. Patients with SDNN values above 100 ms had a 5.3-fold lower risk of mortality than those with an SDNN of 50 ms [18]. In our study, baseline values were reduced in both groups: 45.7 ms in the NW&EB group and 41.7 ms in the G group. After the vacation, only the NW&EB group revealed a significant increase in SDNN (+ 4 ms) (*p* < *0.05*). We assume that a longer vacation would have led to more pronounced changes in HRV in both groups. A study published by Melanson et al. in 2001, which examined the effects of a moderately to highly intensive long-term training program over 12 weeks, reported a significant increase in both, the time and the frequency domain, supports this notion [26].

In our study, the increase in HRV was more pronounced in the NW&EB group than in the G group. The NW&EB group revealed a significant increase in SDNN, while their r-MSSD and pNN50 improved to a greater extent than in the G group. Nordic walking and e-biking are traditional aerobic endurance sports. Golf involves a great deal of dynamic and isometric muscle contractions and is characterized by significantly lower exercise intensity and energy demands (3–4 METs) compared to Nordic walking or e-biking (5–6 METs) [4]. The published literature suggests that HRV is primarily influenced by aerobic exercise of a certain threshold of intensity [10]. The fact that the changes in HRV were not significant in the G group—and only the change in SDNN was significant in the NW&EB group – may be explained by the fact that the required intensity threshold or product of exercise duration and intensity was not achieved in the G group.

The improvements in well-being and autonomic status observed in the present study confirm pre-existing evidence and should encourage the general population to raise their physical activity levels. The data are in line with the current 2020 ESC guidelines on sports cardiology and exercise in patients with cardiovascular disease. The guideline recommends moderately intensive aerobic exercise for at least 150 min per week, spread over five days, for all healthy persons. Alternatively, more intensive training units of 75 min, spread over three days in a week, are recommended to save time in a busy everyday life [27].

Daytime sleepiness and dejection are signs of incomplete regeneration overnight due to poor sleep quality. Altered nocturnal sympathetic activity appears to be the trigger, counteracting physiological parasympathetic predominance during sleep [14]. We derived sleep architecture from the HRV measurements. Although the changes in pNN50 and r-MSSD were not significant, there were similar improvements in both groups on the EBF-24 questionnaire; both groups experienced a reduction of tiredness and dejection. pNN50 and r-MSSD are HRV parameters to analyze autonomic functions, especially parasympathetic activity [14]. Total power and log LF/HF were also slightly increased. These data concur with previous studies, which also demonstrated the effect of exercise (strength training) on HRV in patients with stable coronary heart disease and revealed a significant increase in r-MSSD in the training group [28].

We conclude that both groups showed similar changes in HRV, although some of these did not achieve statistical significance. In combination with the significant results of the questionnaire in regard of well-being, it may be concluded that the exercise program had positive effects on ANS as well.

Hypothesis 2, which postulated positive effects of an active vacation on HRV and ANS, was partly confirmed. Changes in HRV parameters were observed but were not significant with the exception of SDNN in the NW&EB group.

*Ad H3)* Sleep architecture (determined from the HRV) as well as sleep quality on the EBF-24 questionnaire were significantly improved in both groups. Sleep architecture improved from moderately to slightly disturbed sleep in the G group, and from slightly disturbed to nearly normal in the NW&EB group (*p* < *0.05*). Nocturnal PNS activity was significantly increased on the HRV spectrogram (Figs. 1 and 2).

Our results were confirmed in a meta-analysis published by Kredlow et al. in 2016, who investigated the effects of exercise on sleep. Short-term, and especially long-term sporting activities had positive effects on various sleep characteristics, such as total sleep duration, stage 1 sleep, deep sleep, time to fall asleep, and REM sleep. These parameters were dependent on gender, age, basic activity level, duration of activity, type and time of sport exercise [29].

Hypothesis 3, which postulated that the combination of regular exercise and vacation has a positive effect on sleep quality and sleep architecture, was confirmed.

Epidemiological studies showed that an active lifestyle protects the individual from the so-called diseasome of inactivity, and is able to increase life expectancy [30, 31]. Remedies for physical inactivity are one of the foremost public health issues worldwide. Present and future strategies in health policy must be aimed towards increasing physical activity in leisure time and during vacations [32].

A structured training program, as provided by the study design, appears to have learning effects and has a lasting positive effect on health behavior. 85% of the study participants reported long-term changes in their activity behavior and were more health-conscious. The second survey also revealed a notable subjective recovery effect 2–3 months after the vacation.

Although the study design of the OGTS did not differentiate between “activity-related” and “holiday-related” health effects resulting from the sole absence of work stress, we suppose that vacations without an activity program achieve just minor effects on health and well-being recognizing the findings of the meta-analysis by de Bloom et al. in 2009 [7].

## Conclusions

The present study showed that a short-term vacation of seven days with physical activities of low to moderate intensity significantly improves well-being and sleep quality in healthy persons. We also noted positive effects on the ANS and HRV. The inactive lifestyles of modern society are expected to raise the frequency of inactivity-related metabolic, cardiovascular, oncologic, psychiatric and neurodegenerative diseases. This cluster of diseases, also known as the diseasome of inactivity, can be prevented by a more physically active lifestyle in daily life. These will be the prime goal of future public health policies. Information campaigns, education programs, enlightenment and knowledge transfer by mass media will be needed to encourage greater activity in everyday life and during vacations.

The East Tyrol Health Tourism Study is a preliminary step in scientific research on this health-related issue. An active short-term vacation was shown to be a safe, health-enhancing measure and served as an initial spark in achieving a more active lifestyle.

### Limitations

Additional factors which could have affected the outcome of the study, for instance caloric intake during the holidays, the socioeconomic status or job profile of the participants were not considered.

## Data Availability

The datasets used and/or analysed during the current study are available from the corresponding author on reasonable request.

## Abbreviations

HRV: Heart rate variability
ECG: Electrocardiogram
OGTS: East Tyrol Health Tourism Study
NW&EB: Nordic walking and e-biking
WHO: World Health Organization
BMI: Body mass index
SDNN: Standard deviation of the NN intervals in ms
pNN50: Percentage of the intervals with at least 50 ms deviation from the previous interval
r-MSSD: Root mean square of successive differences between normal heartbeats
logLF/HF: Quotient of the logarithm of low frequency to high frequency
Spirit: Standard protocol items: recommendations for interventional trials
